# Preparation of Agrowaste-Based Nanocellulose by NaOH-Assisted Ball Milling Technique: Influence of Component Intervention

**DOI:** 10.3390/gels11080631

**Published:** 2025-08-11

**Authors:** Yuxi Wang, Yong Yu, Shuhan Hu, Jinyao Yu, Yue Huang, Hongjie Dai

**Affiliations:** 1 Yibin Academy of Southwest University, Yibin 644000, China; akuraou1128@163.com (Y.W.); yuyong@swu.edu.cn (Y.Y.); jasmine131419@163.com (J.Y.); 2 College of Food Science, Southwest University, Chongqing 400715, China; 13972605065@163.com; 3 Chongqing Sericulture Science and Technology Research Institute, Chongqing 400700, China; huangyuecn@163.com

**Keywords:** pineapple peel, nanocellulose, component, characterization, ball milling, sodium hydroxide

## Abstract

Pineapple peel cellulose nanofibrils (PCNFs) were facilely prepared by the ball milling method assisted by alkali solution (3 wt% NaOH) and a wet grinding medium, using various treated pineapple peels (hot water treatment (WT), bleaching treatment (BT), alkaline treatment (AT), and baleaching–alkaline treatment (ABT)) as raw materials. The structure of the obtained PCNFs (i.e., WT-PCNF, BT-PCNF, AT-PCNF, and ABT-PCNF) was characterized to analyze the influence of component intervention. The results indicated that NaOH-assisted ball milling did not change the crystal structure of cellulose, and the yield and thermal stability of the PCNFs was improved. The average diameters of WT-PCNF, BT-PCNF, AT-PCNF, and ABT-PCNF were 24.16, 21.53, 23.04, and 19.46 nm, respectively, in which BT-PCNF and ABT-PCNF exhibited a higher defibrillating degree and smaller diameter. Particularly, NaOH-assisted ball milling can promote the removal of non-cellulose components. The viscosity and modulus of BT-PCNF were relatively higher due to the presence of residual hemicellulose as a natural linker of fibers. The current research provides insights for simplifying the preparation and functionalization of nanocellulose.

## 1. Introduction

Recently, the urgent need for sustainable energy sources has extremely promoted the utilization of biomass resources and agricultural wastes [1]. Cellulose, as the most abundant and renewable resources on earth, is considered as a potential alternative to conventional energy and material sources [2,3]. Nanocelluloses, as a nanoscale form of cellulose, exhibit remarkable advantages such as biocompatibility, degradability, a high specific surface area, and mechanical strength, attracting widespread interest in various industrial fields such as food, biomedicine, agriculture, environment, and biosensing devices [3,4,5,6,7,8]. Although nanocelluloses has many benefits, the current preparation method is relatively complex with a high production cost, since it involves the extraction and nanoscale operations of cellulose [2]. The main constituents of lignocellulosic biomass as a complex hierarchical network primarily include cellulose, hemicellulose, and lignin, which are strongly entangled and chemically bonded by non-covalent forces (hydrogen bonds) and covalent cross-linkages, making their separation or isolation challenging [9,10]. For example, the preparation of nanocellulose from lignocellulosic biomass feedstocks usually requires the pre-removals of lignin and hemicellulose to obtain pure cellulose, which could lead to rather cumbersome production steps and resource wastes [11].

Recently, some researchers have confirmed the positive influence of lignin and/or hemicellulose retention on the extraction and properties of nanocellulose from lignocellulosic feedstocks without bleaching and/or alkali treatments, as compared to conventional nanocellulose production [10]. For example, the retention of lignin and/or hemicellulose can improve the performance of nanocellulose, such as surface charge, stability, hydrophobicity, antioxidant activity, UV-blocking ability, and mechanical properties [9,12,13]. In plant cell walls, crystalline cellulose microfibrils are embedded in the network of amorphous hemicelluloses, while lignin hardens the cell wall and provides structural rigidity and physical barrier like the structure of reinforced concrete with a steel core, cement, and buffering material [14]. Therefore, it is challenging to achieve cellulose defibrination and depolymerization under a complete cell wall structure. However, conventional purification and depolymerization of cellulose involve the use of large amounts of toxic and/or expensive reagents (e.g., concentrated strong acids, sodium chlorite, and organic solvents) as well as complex processes, leading to environmental concerns and high costs [2,5,10]. It is imperative to develop an environmental, efficient, and scalable method for the production of nanocellulose.

Nanocellulose, especially cellulose nanofibrils (CNFs), can be usually obtained through a mechanical shearing operation such as homogenization, microfluidization, ultrasonic, and grinding treatments [10]. Notably, some chemical treatments (e.g., acid hydrolysis, oxidation, and enzymatic hydrolysis) can facilitate this process and promote the removals of lignin and hemicellulose during mechanical shearing [15]. Among these different types of mechano-chemical processing of cellulose, ball milling is an emerging technique and has obtained growing interest due to the fact that it is easy to use, requires less expensive equipment, has simple operation steps, is economical, and is environmentally friendly [16]. Recently, ball milling has been explored to obtain CNF under wet conditions, which can promote the swelling of cellulose and accelerate the disruption of hydrogen bonds, avoiding excessive grinding [17,18]. Notably, most of these processes require two steps, namely ball milling after chemical pretreatment, such as TEMPO oxidation [19,20] and deep eutectic solvent [21], and enzymatic treatment [22], which can facilitate the cellulose defibrillation and high purity as well as reduce the cost and energy consumption [23]. However, approaches that synergistically integrate chemical and mechanical actions within a single processing phase remain scarce.

Pineapples have been cultivated on more than a million hectares of soil with the global pineapple production exceeding 28 million tons annually, where approximately 45–55% of processed raw materials can be converted into by-products (e.g., residual pulp, peels, stem, pomace, and crown) [24,25]. In fact, pineapple wastes are the typical case of lignocellulosic biomass that can be utilized as a clean, green, and sustainable sources. Beyond pineapple peel, nanocellulose has been successfully isolated from various agrowastes demonstrating similar lignocellulosic complexity, such as sugarcane bagasse [26], rice straw [27], corn stover [28], and banana peel [29]. Utilizing pineapple peel residues offers significant global availability as an underutilized byproduct. The conventional nanocellulose methods using cellulose as raw material generally involve the use of high-concentration bleaching/alkali treatment reagents for cellulose extraction. In order to reduce production costs and environmental pollution, in this study, a “component-targeted retention and alkali assisted ball milling” strategy was proposed to prepare pineapple peel CNF (PCNF), using pineapple peel residue as raw materials with specific pretreatments (hot water, bleaching, or/and alkaline) for the selective removals of lignin and/or hemicellulose. The goal was to retain selected non-cellulosic components in the starting material, anticipating that these retained components will influence the properties of the resulting nanocellulose (PCNF), such as its morphology, surface chemistry, thermal stability, and rheological behavior, potentially offering advantages over fully purified nanocellulose. In conventional wet ball milling for nanocellulose production, water is commonly used as the grinding medium. We speculated that the use of alkali solution (3 wt% NaOH) as a wet grinding medium could not only promote cellulose swelling and hydrogen bond disruption, but also induce the removal of non-cellulose components. The effect of various treated pineapple peels on the structure and properties of PCNF was investigated.

## 2. Results and Discussion

### 2.1. The Yield Analysis

**Figure 1:**
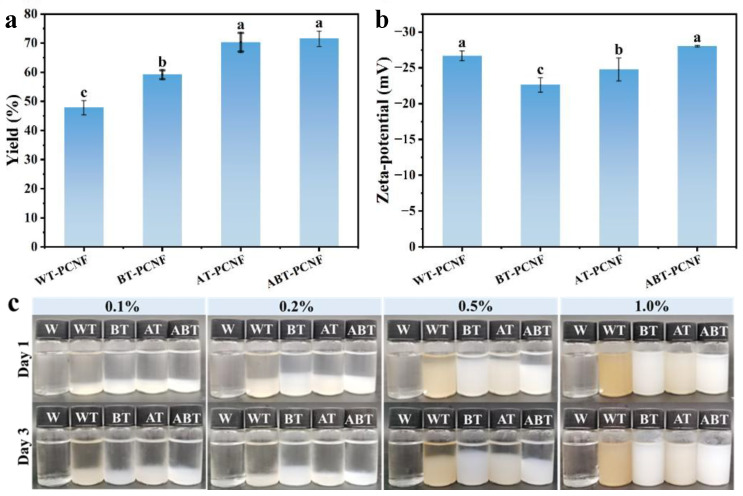
The yield (**a**), zeta potential (**b**) of the obtained PCNF suspensions, and (**c**) appearance change (**c**) of PCNF suspensions with different concentrations after storage for 1 and 3 d at room temperature. Different lowercase letters indicate significant differences between samples (*p* < 0.05).

As shown in Figure 1a, the yield of WT-PCNF, BT-PCNF, AT-PCNF, and ABT-PCNF was 47.9%, 59.2%, 70.3%, and 71.6%, respectively. The yield of PCNF varied significantly with different raw materials. In general, the yield of PCNF increased with the removal of non-cellulose components (lignin and hemicellulose). For example, WT-PP retained both hemicellulose and lignin after simple hot water pretreatment. However, upon treatment with NaOH solution during ball milling, the existed hemicellulose and alkali-soluble lignin could be partially dissolved and subsequently removed along with the supernatant during centrifugation [30]. In addition, due to the relatively compact structure of the unpretreated fibers (WT-PP), the defibrillating effect was inhibited by the presence of lignin and hemicellulose [31]. Most of the fibers are still unable to form nanostructures and exist in the form of precipitation, thus resulting in the lowest yield of WT-PCNF. Compared with WT-PP, BT-PP was bleached to remove most of lignin, and hemicellulose was partial removed by NaOH-assisted ball milling. The loose fibrous structure led to a relatively higher yield of BT-PCNF compared to WT-PCNF. Similarly, AT-PP removed most of hemicellulose and alkali-soluble cellulose but retained alkali-insoluble lignin and cellulose, thus resulting in minimal dissolution loss. Comparatively, the final yield of ABT-PCNF was very close to that of AT-PCNF, which is mainly caused by the higher efficiency of cellulose defibrination, due to the high purity of cellulose raw materials (ABT-PP).

Overall, most of the material loss can occur in the alkali ball milling step of the process [32]. NaOH solution can dissolve hemicellulose and alkali-soluble lignin in pineapple peel residue to a certain extent. Moreover, when combined with the mechanical force of ball milling, it can more effectively degrade the amorphous regions within cellulose, thereby further promoting the depolymerization of cellulose [33]. Compared with hemicellulose, lignin exhibited relatively lower reactivity during alkali treatment, primarily due to its stronger carbon-carbon bonds, aromatic groups, and aromatic rings, which confer a stronger resistance to chemical reactions [34]. In short, using properly treated fiber raw materials and alkaline reagents as ball milling auxiliaries, a higher yield similar to that of traditional cellulose raw materials can also be obtained.

### 2.2. Zeta Potential

As depicted in Figure 1b, the zeta potentials of WT-PCNF, BT-PCNF, AT-PCNF, and ABT-PCNF were −26.67, −22.63, −24.77, and −28.01 mV, respectively. Generally, the absolute value of zeta potential that is greater than 25 mV indicates a relatively stable suspension [34], and values near to 25 mV for all samples were at the boundary between stable colloids and unstable colloids, representing the possible tendency of flocculation and agglomeration. BT-PCNF and AT-PCNF showed relatively low zeta potential values, which may be due to the current dissolution of non-fiber components such as lignin and hemicellulose through bleaching or alkaline treatment, thus not promoting sufficient electrostatic repulsion between fibers [35]. However, the largest values gained for ABT-PCNF were probably related to the fiber size. The visual appearance of PCNF suspensions is shown in Figure 1c. WT-PCNF and AT-PCNF appear as brown-orange due to unremoved lignin. In addition, it is apparent that the PCNF suspensions are unstable, especially at low concentrations (<1.0%). This can be more clearly observed through the formation of sediment when the suspensions were left to rest for 72 h.

### 2.3. FTIR Analysis

**Figure 2:**
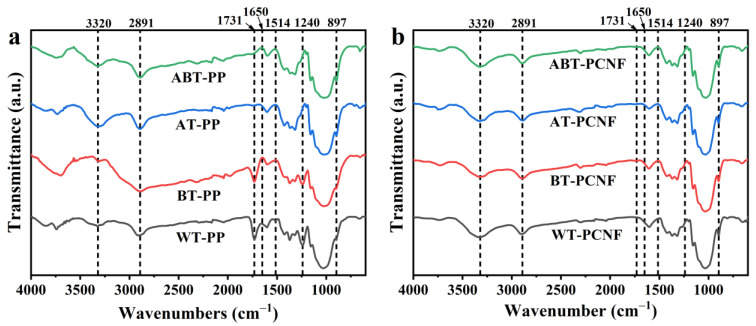
FTIR spectra of the treated PP (**a**) and the obtained PCNF (**b**).

FTIR spectra of the treated PP and the obtained PCNF are shown in Figure 2a and b, respectively. All the samples show some typical characteristic peaks representing cellulose. The absorption peak at 1650 cm^−1^ is attributed to the -OH bending vibration of natural bound water [36], the peaks at 3320 and 2891 cm^−1^ indicate the O-H stretching and C-H bond deformation on the cellulose framework [37], and the absorption peak at 897 cm^−1^ corresponds to glycoside C-H deformation with O-H bending, representing the presence of β-glycosidic linkages in cellulose [38]. Notably, all samples show the characteristic peaks of a cellulose I structure, indicating that the basic structure of cellulose after various pretreatments and ball milling was not destroyed. After pretreatment, only the characteristic peak at 1731 cm^−1^ is observed in the spectrum of WT-PP and BT-PP, attributed to the acetyl and uronic ester groups in the hemicelluloses, which may also be caused by the ester linkage between ferulic acids and p-coumaric acids of lignin and/or hemicelluloses [39]. The peaks at 1514 and 1240 cm^−1^ are observed in WT-PP and BT-PP, respectively, representing the -C=C- stretch of aromatic rings and =C-O- stretch vibration in lignin [40]. After the subsequent dilute alkaline–ball milling treatment for nanocellulose preparation, the spectrums of the PCNF sample are similar, the characteristic peaks generated by the basic structure of cellulose are successfully maintained, and the intensity of absorption peaks of lignin and hemicellulose decrease or disappear (especially in 1731 cm^−1^ and 1240 cm^−1^), implying that non-cellulose components in the raw materials were mostly removed through dilute alkaline–ball milling treatment, which is beneficial to improve the purity of nanocellulose. While the FTIR spectra of the final PCNFs (Figure 2b) appear largely similar, reflecting the predominant cellulose I structure, the key distinction imparted by the pretreatment lies not in introducing new functional groups to the final cellulose core, but in determining the extent of the non-cellulosic component removal achieved during the NaOH-assisted ball milling process. This differential removal, as evidenced by the varying intensities of residual peaks, is the primary mechanism through which pretreatment dictates the properties of the resulting PCNFs.

Further insights into the structure of the PCNFs were obtained by calculating the infrared crystallinity ratio (R). The calculated R values were 0.9454 for WT-PCNF, 1.0058 for BT-PCNF, 0.9904 for AT-PCNF, and 0.9906 for ABT-PCNF. This ratio generally increases with higher cellulose crystallinity. While WT-PCNF showed the lowest R value, consistent with the presence of more amorphous components hindering the crystalline packing, BT-PCNF exhibited the highest R value. ABT-PCNF and AT-PCNF showed intermediate R values, aligning with their respective compositions and the effectiveness of the combined treatments in enhancing crystalline order.

### 2.4. Light Transmittance Analysis

**Figure 3:**
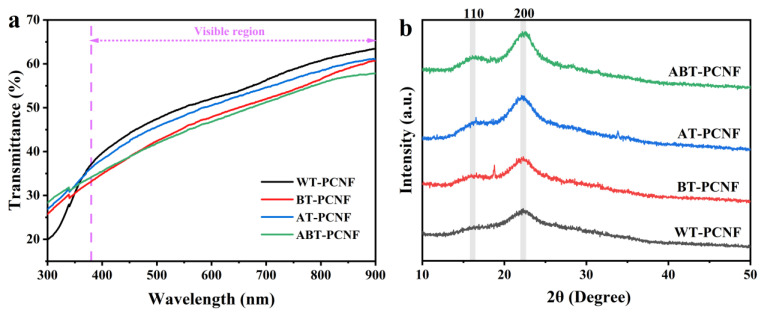
Light transmittance (**a**) and XRD patterns (**b**) of the obtained PCNF.

The light transmittance of obtained PCNF suspensions within the wavelength range of 300–900 nm is shown in Figure 3a. In the visible region range, the transmittance of PCNF decreases in the order of WT-PCNF, AT-PCNF, BT-PCNF, and ABT-PCNF, and the transmittance at 600 nm is 52.0%, 50.1%, 49.0%, and 46.7%, respectively. In contrast, ABT-PCNF exhibits a relatively lower transparency because light transmittance is closely related to the crystallinity of nanocellulose. The non-cellulosic components (lignin and hemicellulose) of ABT-PCNF are mostly removed by alkaline and bleaching treatments to obtain nanocellulose with higher crystallinity [41], and its molecular structure is arranged in a more orderly manner, which makes it easier to reflect incident light when it illuminates the surface of fibers, thus reducing the light transmission of the material [42].

### 2.5. XRD Analysis

**Figure 4:**
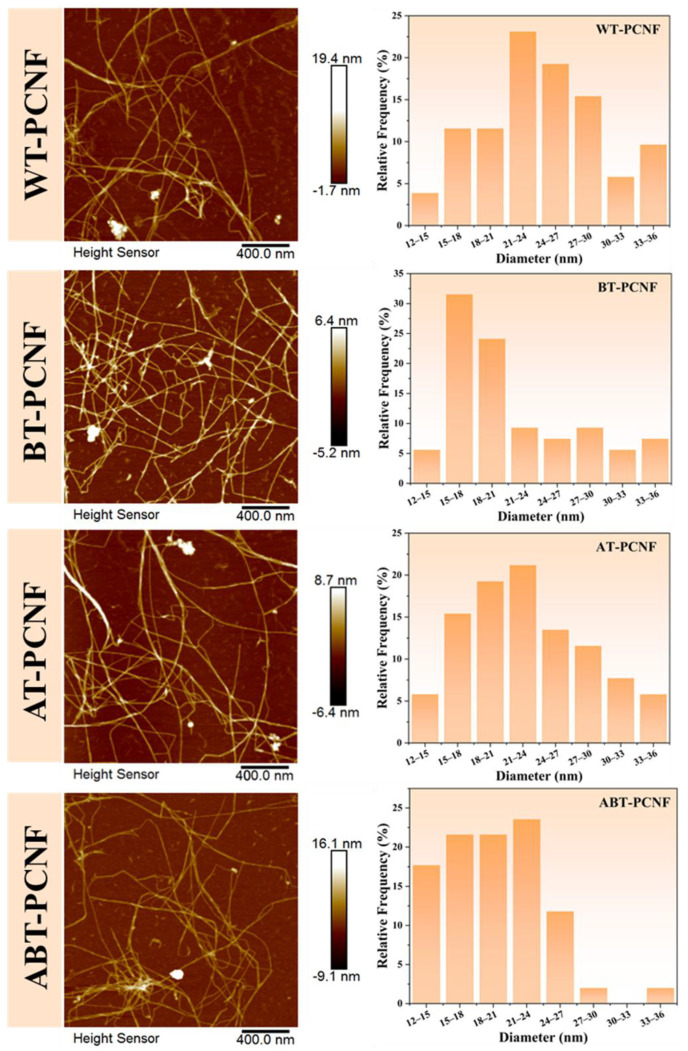
AFM diagram of the obtained PCNF.

Figure 3b shows the XRD patterns of all PCNF samples. The crystalline peaks at 2θ angles of 16.4° and 22.5° correspond to the cellulose principal crystal plane (110) and (200), respectively, which are typical spectrums of the cellulose I structure [43]. The results show that the cellulose structure of PCNF did not change and maintained favorable crystal morphology. According to the Segal formula, the CrI value of WT-PCNF, BT-PCNF, AT-PCNF, and ABT-PCNF was 38.4%, 38.0%, 41.8%, and 44.2%, respectively. Alkaline and bleaching treatments can effectively remove most of the hemicellulose and lignin in pineapple peel residue, resulting in the rearrangement of cellulose molecules and the improvement of the crystallinity of PCNF [44]. ABT-PCNF had the highest crystallinity due to the removal of the amorphous zone by alkali/bleaching pretreatments and dilute alkaline-assisted ball milling treatment. Notably, the crystallinity of BT-PCNF was slightly lower than that of WT-PCNF, possibly attributed to the high concentration of NaClO_2_ during delignification by bleaching, resulting in slight destruction of the crystalline zone [45]. The crystallinity trends are consistent with the structural insights gained from the FTIR analysis. The highest CrI for ABT-PCNF corresponds to its high R value, confirming its superior crystallinity. The slightly lower CrI of BT-PCNF compared to WT-PCNF, despite BT-PCNF having the highest R value, likely reflects differences in technique sensitivity: XRD probes long-range lattice order, while the FTIR ratio may be more sensitive to shorter-range molecular interactions potentially enhanced by bleaching. The calculated crystal size D values for WT-PCNF, BT-PCNF, AT-PCNF, and ABT-PCNF were 2.66, 2.78, 2.49, and 3.00 nm, respectively. These D values represent the average size of the coherently diffracting crystalline domains within the nanofibrils and are significantly smaller than the overall fibril diameters measured by AFM (Section 2.6, Figure 4). This confirms that each PCNF consists of multiple crystalline regions embedded in an amorphous matrix. The variation in the D values suggests that different pretreatments influence the nanoscale crystalline domain structure, with ABT-PCNF showing the largest crystalline domains despite undergoing the most intensive treatment.

### 2.6. AFM Analysis

AFM images of WT-PCNF, BT-PCNF, AT-PCNF, and ABT-PCNF are shown in Figure 4. It can be observed that all the samples exhibit a typical slender filamentary structure of the traditional CNF, and a certain degree of entangled network is formed among these fibers. Notably, some spherical nanoparticles adhering to the fiber network are most likely lignin [46]. Overall, BT-PCNF and ABT-PCNF show minimal particle aggregates due to having the highest removal of lignin. The average diameters of WT-PCNF, BT-PCNF, AT-PCNF, and ABT-PCNF are 24.16, 21.53, 23.04, and 19.46 nm, respectively. Among them, WT-PCNF and AT-PCNF have a lower defibrillating degree and larger diameter, which may be related to the presence of lignin that can inhibit fibrillation of cellulose. As reported, lignin has stable free radicals that protect it during the NaOH-assisted ball milling process and act as a bonding material between fibers, making it more difficult to deconstruct nanocellulose [47]. However, BT-PCNF and ABT-PCNF exhibit a higher defibrillating degree and smaller diameter because lignin in the feedstock is effectively bleached away, which can also be seen in the color of their suspensions (Figure 1c). The results indicate that the morphology of PCNF can be controlled by various pretreatments that can dominate the components in raw materials.

### 2.7. TGA Analysis

**Figure 5:**
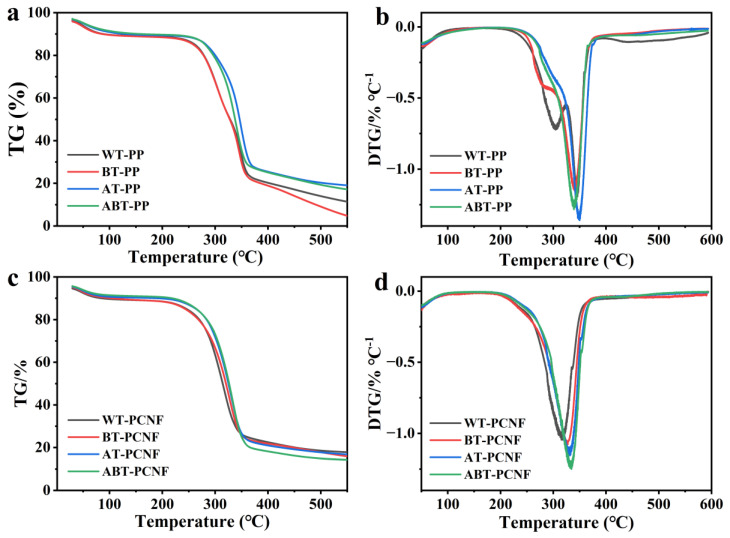
TG (**a**,**c**) and DTG (**b**,**d**) curves of the treated PP and the obtained PCNF.

TGA was conducted to determine the thermal stability and degradation properties of the treated PP and the obtained PCNF. The results were plotted as thermogravimetric (TG) and derivative thermogravimetric (DTG) curves, which are depicted in Figure 5. In general, the major decomposition of the pineapple peel fibers occurred in several stages, implying that the decomposition temperature of various components was different. Figure 5a,c shows an initial weight loss found in PP and PCNF within the region of 30–120 °C, which suggests the evaporation of water molecules physically absorbed on the fibers or bonded into the fibers [48]. The second weight loss step corresponds to the low decomposition temperatures of hemicelluloses. In this stage, PP and PCNF begin to degrade from around 220 °C, and their DTG peaks appear at about 340 °C, which is the main stage of mass decomposition due to the cellulose pyrolysis [39]. As summarized in Table 1, the onset of decomposition temperatures (T_onset_) of WT-PP, BT-PP, AT-PP, ABT-PP, WT-PCNF, BT-PCNF, AT-PCNF, and ABT-PCNF is 223.5, 234.3, 236.5, 239.9, 232.0, 237.3, 240.5, and 245.1 °C, respectively. Notably, the T_onset_ of the obtained PCNF is higher than that of the treated PP, indicating that PCNF prepared by NaOH-assisted ball milling is more thermally stable than cellulose in raw lignocellulosic biomass due to its higher crystallinity, flexible structure, and removal of non-cellulose components with low thermal stability [49]. AT-PP and ABT-PP show high residues, attributed to alkali-insoluble lignin that forms stable char. BT-PP has the lowest residue, reflecting extensive delignification by bleaching. PCNF residues are higher than BT-PP but lower than lignin-rich WT-PP/AT-PP, aligning with partial lignin removal during ball milling. As depicted in Figure 5b, in the case of WT-PP and BT-PP, a main DTG peak is found at 345.7 and 340.5 °C, respectively, followed by another shoulder at 304.2 and 285.1 °C, respectively, which could be attributed to the thermal decomposition of hemicellulose and cellulose. However, for other samples, only the thermal decomposition peak of cellulose is observed in their DTG curves due to the extremely low hemicellulose content. As shown in Figure 5d, the maximum decomposition temperature (T_max_) of WT-PCNF, BT-PCNF, AT-PCNF, and ABT-PCNF increases successively, indicating the successively increased thermal stability. When the temperature is higher than 400 °C, the weight loss is mainly due to the decomposition of lignin, which can be decomposed in a wide range of 100–900 °C [45]. Particularly, after preparing PCNF by NaOH-assisted ball milling, the shoulder peak originally in WT-PP and BT-PP basically disappears, which is mainly attributed to the dissolution of hemicellulose assisted by NaOH during the ball milling process. Overall, ABT-PCNF obtained from pure cellulose (ABT-PP) displays better thermal stability, but PCNF isolated from the raw materials retaining other components (WT-PP, AT-PP, and BT-PP) can also achieve good thermal stability by ball milling assisted with alkali solution.

### 2.8. XPS Analysis

**Figure 6:**
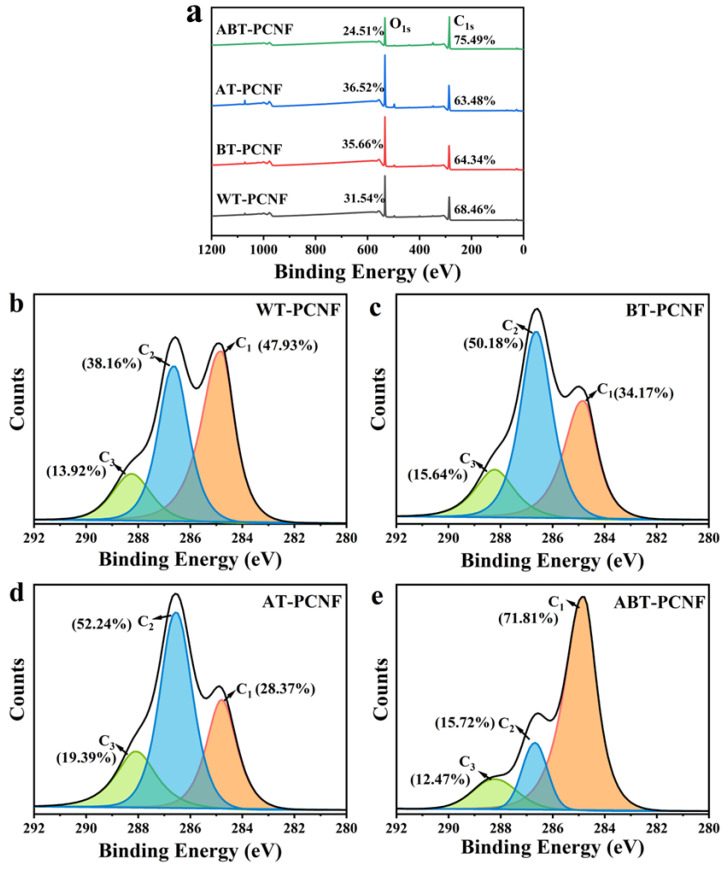
XPS spectra (**a**) and C1s characteristic peaks (**b**–**e**) of the obtained PCNF.

XPS was employed to investigate the surface chemical composition of PCNF. As shown in Figure 6a, the spectra of all PCNF samples exhibit characteristic peaks at 285 eV (C1s) and 532 eV (O1s), with no detectable significant signals from impurity elements, indicating the high purity of the obtained materials. Quantitative analysis revealed distinct oxygen-to-carbon (O/C) ratios of 0.46, 0.55, 0.57, and 0.32 for WT-PCNF, BT-PCNF, AT-PCNF, and ABT-PCNF, respectively. Compared to WT-PCNF, the increased O/C ratio observed in BT-PCNF and AT-PCNF is attributed to more exposure of hydroxyl groups through intensified defibrillation during ball milling [50]. However, the significantly reduced O/C ratio observed in ABT-PCNF could be attributed to the removal of partial hemicellulose induced by comprehensive pretreatment protocol involving multiple stages.

High-resolution C1s spectra (Figure 6b–e) were deconvoluted into three characteristic components: C-C/C-H (284.80 eV), C-O (286.70 eV), and O-C-O/C=O (288.30 eV) [51], corresponding to the C1, C2, and C3 positions in the cellulose molecular chain, respectively. Notably, the relative content of C-O components exhibited a positive correlation with O/C ratio increments, providing direct evidence for differential hydroxyl group exposure. The adjustable surface properties facilitate controlled chemical modifications focused on hydroxyl groups. Despite some structural modifications induced by pretreatment process, XPS analysis confirmed the preservation of chemical signatures associated with the cellulose I crystalline structure. This observation aligns well with XRD patterns (Figure 2b), demonstrating that the applied ball milling strategy effectively maintained the intrinsic chemical identity of cellulose during nano-structuring. Both the XPS and XRD results highlight the advantage of component-controlled pretreatment in achieving nanoscale defibrillation without compromising cellulose’s essential chemical characteristics.

### 2.9. Rheological Analysis

**Figure 7:**
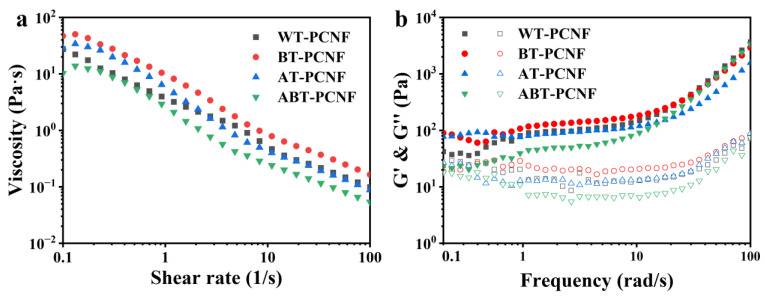
Shear rate–viscosity curve (**a**) and dynamic frequency sweep curve (**b**) of the obtained PCNF; solid symbols represent storage modulus (G′); open symbols represent loss modulus (G″).

The shear rheological behavior of PCNF suspensions is illustrated in Figure 7a. At low shear rates, all PCNF samples exhibit high apparent viscosity, which progressively decreases with increasing shear rate, demonstrating typical shear-thinning behavior [52]. The rheological properties of PCNF suspensions were mainly affected by the concentration of PCNF and the network structure composed of PCNF. Due to the strong network formed by the high entanglement between nanocellulose, these long and entangled fibers can effectively connect to each other, forming stable network structure with gelation properties, even at lower concentration conditions. Due to shear forces, the network structure is destroyed and the slender nanofibers move, resulting in a decrease in viscosity [52]. Notably, ABT-PCNF shows the lowest viscosity values at equivalent shear rates, which is closely related to its morphological characteristics. The bleaching and alkali treatments reduce the particle size and surface modification of the fibers, thereby resulting in a decrease in the physical entanglement density between fibrils and consequently weakening shear resistance of the network structure.

The dynamic frequency scanning of PCNF is shown in Figure 7b. Within the angular frequency range of 0.1–100 rad/s, the storage modulus (G′) of all samples exceeds the loss modulus (G″), indicating a typical gel-like behavior dominated by their elastic properties. The G′ and G″ of the samples initially decrease and then increase with the increase in angular frequency. The slight decrease at a low frequency corresponds to the partial relaxation of the network structure, while the increase in modulus at a high frequency reflects the rapid rearrangement of fiber segments under strong shear [53]. Overall, BT-PCNF displays the highest G′ and G″ values, indicating that the elasticity and viscosity of the PCNF suspensions are enhanced significantly by bleaching alone, which is related to the size and quantity of fibers in the system. The hemicellulose retained in BT-PCNF could enhance the rheological properties of the system as a natural linker of fibers. This observation shows that the rheological properties of PCNF can be achieved by changing the composition of the pretreated feedstocks used for ball milling.

## 3. Conclusions

The structurally controllable PCNF was facilely prepared by NaOH-assisted ball milling using pineapple peel residue under different pretreatments as raw materials. Unlike the traditional preparation of nanocellulose that requires the extraction of pure cellulose through bleaching and alkali treatments, the current research demonstrated that stable PCNF can be isolated using the ball milling process without treatment or with moderate treatment of pineapple peel. XRD and FTIR results showed that the cellulose Iβ crystal structure of PCNF was not damaged after different pretreatments (alkali and/or bleaching treatment) combined with alkali-assisted ball milling. The differential extraction of non-cellulose components was identified as the pivotal factor governing PCNF performance. ABT-PCNF showed the highest yield, simultaneously reducing fibril diameter while delivering the highest crystallinity and thermal stability. BT-PCNF can preferentially remove lignin, exposing additional hydroxyl groups and enhancing interfibrillar hydrogen bonding to achieve maximum storage modulus. In contrast, WT-PCNF and AT-PCNF containing lignin exhibited larger diameters and higher transmittance due to lignin’s radical-protection effect that inhibits defibrillation. Taking into account the cost and energy consumption differences among various pretreatment methods and the performance of the final nanocellulose products (especially in color, morphology, and rheological properties), BT-PCNF can achieve comparable properties to ABT-PCNF obtained from the raw material after the traditional bleaching–alkaline treatments. This can serve as a potential method for subsequent industrial production.

## 4. Materials and Methods

### 4.1. Materials and Reagents

The fresh pineapple peel residue was freely provided by local fruit supermarkets in Chongqing (China). Sodium chlorite (NaClO_2_) was provided by Yuexiang Chemical Co., Ltd. (Chongqing, China). Sodium hydroxide (NaOH), hydrochloric acid (HCl), and ethanol were obtained from Chron Chemical Reagent Co., Ltd. (Chengdu, China). Potassium bromide (KBr) was purchased from Aladdin Biochemical Technology Co., Ltd. (Shanghai, China). All other chemical reagents were of analytical grade without further purification.

### 4.2. Component Regulation of Pineapple Peel Residue

The component regulation of pineapple peel residue was carried out according to our previous study [44]. Briefly, the collected pineapple peel residue was rinsed in clean water and crushed by a pineapple juice extractor, followed by filtration to collect the filtration residue. The residue was washed with clean water until the rinse solution was colorless. After being dried at 60 °C for 24 h and pulverized, the powdered pineapple peel residue (PP) was obtained for further use. In order to extract nanocellulose from raw materials containing different components, PP was treated by following steps that are common in cellulose purification processes prior to traditional nanocellulose extraction.

Hot water-treated PP (WT-PP): Briefly, PP was suspended with distilled water at a solid–liquid ratio of 1:20 (g/mL) and stirred at 80 °C for 2 h to remove water-soluble impurities. Then, the treated PP was filtered to collect a solid fraction, followed by washing with distilled water. After being dried and pulverized again, the obtained WT-PP was available for further use.

Bleaching-treated PP (BT-PP): Briefly, WT-PP was mixed with NaClO_2_ solution (7.5 wt%, pH 3.8–4.0) at a solid–liquid ratio of 1:20 (g/mL) to remove lignin (delignification). The bleaching process was maintained at 75 °C for 5 h. Afterwards, the residual solids were repeatedly washed with distilled water until the pH of the filtrate was close to neutral. After being dried and pulverized again, the obtained BT-PP was available for further use.

Alkaline-treated PP (AT-PP): Briefly, WT-PP was mixed with NaOH solution (10 wt%) at a solid–liquid ratio of 1:20 (g/mL) to remove hemicellulose and alkali-soluble lignin. The mixture was stirred at room temperature for 10 h. After being filtered and washed with distilled water, the residual solids (AT-PP) were dried and pulverized for further use.

Bleaching–alkaline-treated PP (ABT-PP): The preparation process of ABT-PP was basically the same as that of AT-PP; only the raw material (WT-PP) was replaced with BT-PP.

### 4.3. Preparation of PCNF

The preparation of PCNF was performed by a facile ball milling technique using 3 wt% NaOH solution as the ball milling assistant [54]. Briefly, 1.5 g of the samples (WT-PP, BT-PP, AT-PP, and ABT-PP) dispersed in 3% NaOH solution was placed in a zirconia vessel containing zirconia balls (90 g) of different diameters (15, 12, 10, 8, and 5 mm). The ball milling was conducted using a vertical planetary ball mill (XQM-0.4A, Tencan powder, Changsha, China). The parameters of the ball milling process were based on our previous research, showing a speed of 400 rpm for 3 h in cycles of 20 min/milling per 10 min/rest at room temperature [20]. This milling regimen can achieve sufficient nanofibrillation while minimizing excessive energy input, potential overheating, and amorphous region degradation that could occur with continuous high-intensity milling. The intermittent rest periods aid in heat dissipation. Afterwards, the resulting slurry was centrifuged (5000 r/min, 10 min) and washed twice with distilled water, followed by dialysis (using cellulose ester dialysis membranes with a molecular weight cut-off of 12–14 kDa) against distilled water at room temperature for 72 h to remove degradation products and residual NaOH. The water was changed every 4–8 h. The obtained suspensions were further dispersed by ultrasonic treatment (300 W, 30 min) and stored at 4 °C for further use or freeze-drying for characterization. According to the difference of raw materials, the obtained PCNFs were coded as WT-PCNF, BT-PCNF, AT-PCNF, and ABT-PCNF, respectively. The yield of the PCNF was determined by directly drying PCNF suspensions at 60 °C until no decrease in mass was detected; then, it was calculated using the following equation [20]:

(1) $$\mathrm{Yield}\;(\%)\;=\;\frac{M_{1}}{M}\times 100$$

where *M* is the initial weight of PP (g), and *M*_1_ is the weight of PCNF (g) after drying at 60 °C.

### 4.4. Characterization of PCNF

#### 4.4.1. Zeta Potential

The zeta potentials of each PCNF suspensions were measured using a Zetasizer Nano ZS 90 (Malvern Instruments Ltd., Malvern, UK) at 25 °C. All PCNF suspensions were diluted with distilled water to a concentration of 0.05 wt% and ultrasonicated for 5 min at 300 W. The zeta potential data points were the average values of 11 measurements.

#### 4.4.2. Optical Transmittance

The optical transmittance of each PCNF suspension (0.05 wt%) was recorded at a wavelength range of 300 to 900 nm using a UV–vis spectrophotometer (UV 6100, Metash, Shanghai, China). The transmission spectra were recorded using distilled water as a reference to correct the transmittance of the suspensions at room temperature [55].

#### 4.4.3. Atomic Force Microscope (AFM)

AFM mapping of PCNF was performed using an atomic force microscope (Dimension Icon, Bruker, Germany). Scanning was performed in tapping mode using a silicon cantilever at a scan rate of 1.0 Hz. Prior to analysis, the diluted PCNF suspensions (0.01 wt%) were ultrasonicated for 10 min to ensure uniform dispersion, then a droplet was deposited on the surface of freshly cleaved mica and dried at room temperature for observation. The average value and distribution of the diameter were statistically analyzed using the Nano Measure 1.2 software.

#### 4.4.4. Fourier Transform Infrared Spectroscopy (FTIR)

FTIR spectra of the initial raw materials (i.e., WT-PP, AT-PP, BT-PP, and ABT-PP) and the obtained PCNF (i.e., WT-PCNF, BT-PCNF, AT-PCNF, and ABT-PCNF) were collected using a Fourier transform infrared spectrometer (Spectrum Two, PE, Waltham, MA, USA). The instrument was calibrated for wavenumber accuracy using a polystyrene film standard prior to sample measurements. The spectra data were recorded in the wavenumber range of 4000–600 cm^−1^ by accumulating 32 scans at a resolution of 4 cm^−1^. The infrared crystallinity ratio (R) was calculated as the ratio of the absorbance peak heights at 1372 cm^−1^ and 2900 cm^−1^ [56].

#### 4.4.5. X-Ray Diffraction (XRD)

The crystalline structure of the obtained PCNF was measured by an X-ray diffractometer (X’Pert3 Powder, Malvern Panalytical, Almelo, The Netherlands) with Cu-Kα radiation (λ = 0.1544 nm) at 40 kV and 40 mA over the angular 2θ range of 10–50° with a scan speed of 4°/min. The instrument was calibrated for angle and intensity using the NIST SRM 640c silicon powder standard prior to measurements. The crystallinity index (CrI) was calculated according to the peak intensity method [57]:

(2) $$\mathrm{CrI}\;(\%)\;=\;\frac{I_{200}-I_{am}}{I_{200}}\times 100$$

where *I*_200_ is the maximum intensity of the (200) plane diffraction peak at 2θ = 22.5°, and *I_am_* is the minimum diffraction intensity of the amorphous region between a (200) and (110) plane reflection at 2θ = 18.5°. The mean crystal size along the (200) plane was estimated using the Scherrer equation [58]:

(3) $$\mathrm{Crystal}\;\mathrm{size}\;D\;=\;\frac{k\lambda }{\beta cos\theta }$$

where k is the Scherrer constant (0.9), *λ* is the X-ray wavelength (0.1544 nm), *β* is the full width at half maximum of the (200) diffraction peak, and *θ* is the Bragg angle.

#### 4.4.6. Thermogravimetric Analysis (TGA)

The thermal behavior of the obtained PCNF was investigated using a thermogravimetric analyzer (TGA550, TA Instruments, New Castle, DE, USA) with a temperature range of 30 to 600 °C at a constant heating rate of 10 °C/min under a N_2_ flow of 50 mL/min. During the thermos decomposition process, the sample weight was continuously recorded as a function of the temperature and time. A derivative (DTG) curve was used to describe the weight loss of the sample per unit time with respect to the temperature.

#### 4.4.7. X-Ray Photoelectron Spectroscopy (XPS)

XPS spectra of the obtained PCNF were recorded using a K-Alpha+TM XPS system (Thermo Fisher Scientific, Waltham, MA, USA) with monochromatic radiation Al Kα (hv = 1486.6 eV) and a pass energy of 100 eV. The samples in powder form were analyzed primarily to identify oxygen and carbon signals. The high resolutions of the C 1s spectra (280~292 eV) and O 1s spectra (522~542 eV) had a step size of 0.05 eV and a pass energy of 50 eV.

#### 4.4.8. Rheological Analysis

The rheological properties of the obtained PCNF suspensions (0.5 wt%) were measured at 25 °C with an Anton Paar MCR 302 rheometer (Graz, Austria) equipped with a parallel-plate configuration (plane diameter 25 mm). The shear viscosity and dynamic shear properties indicted by storage modulus (G′) and loss modulus (G″) were measured. The shear rates (0.1~100 s^−1^) were used to measure steady-state shear viscosity. The dynamic properties were measured at frequency sweeps (0.1~100 rad/s), and the strain amplitude was controlled at 0.1% to ensure the test was within the linear viscoelastic region of the material.

### 4.5. Statistical Analysis

All measurements were repeated at least in triplicate and were partially presented as mean ± standard deviation (SD). Statistical significance was determined by one-way analysis of variance (ANOVA) at the significance level of *p* < 0.05 using SPSS Statistics 26 software.

## Figures and Tables

**Table 1:** Thermal degradation parameters of PP and corresponding PCNF.

Sample	T_onset_ (°C)	T_max_ (°C)	Char Residue at 600 °C (%)
WT-PP	223.5	345.7	11.5
BT-PP	234.3	340.5	4.8
AT-PP	236.5	349.3	19.4
ABT-PP	239.9	339.2	17.4
WT-PCNF	232.0	316.3	17.9
BT-PCNF	237.3	326.4	16.0
AT-PCNF	240.5	330.5	17.1
ABT-PCNF	245.1	333.2	14.5

## Data Availability

The datasets used and/or analyzed during the current study are available from the corresponding author on reasonable request.
